# Characterization of the complete mitochondrial genome of *Laomedia astacina* De Haan, 1841 (Decapoda: Laomediidae) and its phylogenetic implications

**DOI:** 10.1080/23802359.2022.2054374

**Published:** 2022-03-22

**Authors:** Dong Guo, Dandan Zhang, Zhong Tu, Lun Song, Jing Dong, Weikuang Wang

**Affiliations:** a Liaoning Ocean and Fisheries Science Research Institute, Dalian, China; b Department of Environmental Engineering and Science, Feng Chia University, Taichung, Taiwan; c Key Laboratory of Mariculture & Stock Enhancement in North China’s Sea, Ministry of Agriculture, Dalian Ocean University, Dalian, China; d Shandong Fisheries Development and Resource Conservation Center, Yantai, China

**Keywords:** Mitogenome, Laomediidae, *Laomedia astacina*

## Abstract

In this study, we determined the complete mitochondrial genome (mitogenome) of *Laomedia astacina* De Haan, 1841 using next-generation sequencing technology. The total length of the mitogenome sequence of *L. astacina* is 14,795 base pairs, including 13 protein-coding genes (PCGs), 22 transfer RNA genes, and two ribosomal RNA genes. The overall composition of the mitogenome is estimated to be 35.3% A, 38.0% T, 13.8% C, and 12.9% G, indicating that the *L. astacina* mitogenome is rich in A + T (73.3%). The phylogenetic relationships of 13 decapod species were constructed based on the 13 PCGs by the maximum-likelihood approach using IQtree software.

*Laomedia astacina* (Decapoda: Laomediidae) inhabits the northern parts of the Yellow Sea and Bohai Sea of China. Studies of *L. astacina* are rare, with only a few focused on its cheliped morphometry and sexual dimorphism (e.g. Itani and Uchino 2005), and little is known about its phylogenetic position (Tsang et al. 2014). Therefore, in this study, we determined the complete mitochondrial genome (mitogenome) of *L. astacina* (GenBank accession no. MW687595). Our findings provide useful information for further studies of population genetics and phylogeny of *L. astacina*.

*L. astacina* individuals were collected from the Dalian Sea area, Liaoning Province, China (39°12′25″ N, 124°45′27″ E). The total genomic DNA was extracted from one muscle using a modification of the standard phenol–chloroform procedure as described by Li et al (2002). The complete mitogenome of *L. astacina* was sequenced on an Illumina HiSeq 2500 platform (San Diego, CA) and paired-end reads were generated. The gene annotation was performed following the methods described by Zhu et al. (2020) and using online software (GeneMarker, tRNAscan, ORFfinder). At present, the *L. astacina* specimens are stored at the Key Laboratory of Mariculture & Stock Enhancement in North China’s Sea, Ministry of Agriculture and Rural Affairs, Beijing, PR China, Dalian Ocean University, Dalian, China (www.dlou.edu.cn, contact person: Dr. Hao, email: haozhenlin@126.com) under voucher numbers DLOU-KLM-SSH177 to DLOU-KLM-SSH186.

The total length of the *L. astacina* mitogenome is 14,795 base pairs, and the base composition is estimated to be 35.3% A, 38.0% T, 13.8% C, and 12.9% G. The complete mitogenome of *L. astacina* contains 13 protein-coding genes (PCGs), two ribosomal RNA genes, and 22 transfer RNA genes. Most of the genes are encoded on the plus strand, although two ribosomal RNAs (*rrnL* and *rrnS*), four PCGs (*nd1*, *nd4*, *nd4l*, and *nd5*), and seven transfer RNAs (*trnF*, *trnH*, *trnP*, *trnI*, *trnQ*, *trnC*, and *trnY*) are encoded on the minus strand. Among the 13 PCGs, all start with the conventional ATN codon, with ATA for *atp8*, *nd1*, *nd5*, and *cytb*; ATG for *atp6*, *cox1*, *cox3*, *nd2*, and *nd4*; and ATT for *cox2*, *nd3*, and *nd6*. The most common stop codon is TAA, although the stop codon for *nd4* is TAG.

We used the 13 PCGs and a total of 12 decapod species to resolve the phylogenetic position of *L. astacina* using maximum-likelihood analysis. *Longpotamon kenliense* from the Infraorder Brachyura was selected to root the phylogenetic tree (Figure 1). *L. astacina* grouped together with the clade formed by species from the family Upogebiidae, but this grouping was only weakly supported, possibly due to an insufficient phylogenetic signal in the dataset. In future studies, the use of a larger data matrix may resolve the phylogenetic position of *L. astacina* with higher resolution.

**Figure 1.**
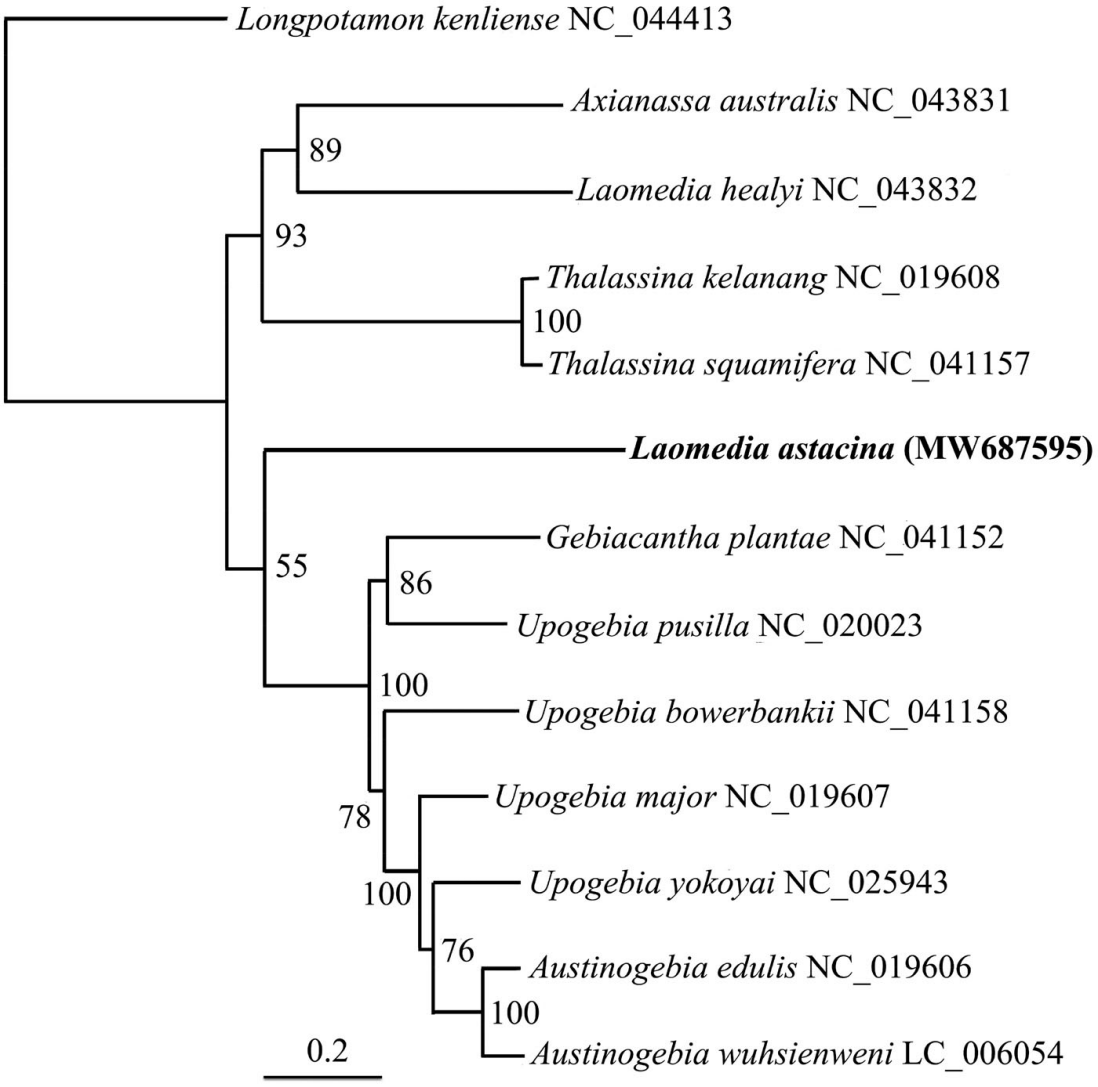
The phylogenetic position of *L. astacina* was inferred using maximum-likelihood analysis based on 13 PCGs in IQ-TREE v1.6.1 (Nguyen et al. 2015). The maximum-likelihood searches were run using a combination of rapid hill-climbing approaches and the stochastic perturbation method with 1000 ultrafast bootstraps.

We expect that our results will contribute to molecular identification of this species and be helpful for exploring the phylogeny of Decapoda.

## Data Availability

The data that support the findings of this study are openly available in GenBank of NCBI at https://www.ncbi.nlm.nih.gov under the accession number MW687595 or are available from the corresponding author. The associated BioProject, SRA, and Bio-Sample numbers are PRJNA753924, SRR15646764, and SAMN21013472, respectively.
